# Editorial: Exploring neuroinflammatory pathways that contribute to chronic pain, volume II

**DOI:** 10.3389/fphar.2024.1426157

**Published:** 2024-06-10

**Authors:** Lisa A. Lione, Amy Fisher

**Affiliations:** ^1^ University of Hertfordshire, Hatfield, United Kingdom; ^2^ GW Pharmaceuticals, Cambridge, United Kingdom

**Keywords:** neuroinflammation, neuropathic pain, STAT2, PPAR γ, CX3CR1, progesterone, ncRNA

Worldwide, health problems caused by chronic pain is escalating with approximately 10% of adults diagnosed each year and prevalence increasing with age, affecting 30% of older adults (Vos et al., 2017). There is a huge unmet need for new effective therapies for the management and/or prevention of multifarious chronic pain conditions. Treatment options are limited and often fail to achieve satisfactory results in part due to the variety of chronic pain conditions with different aetiologies (e.g., diabetes, cancer, viral, musculoskeletal) and complex pathophysiological mechanisms.

Neuroinflammatory cellular and molecular immune components including microglia and astrocytes, cytokines, complement, and pattern-recognition receptors act as key regulators of pain signalling. The exact mechanisms underlying the link between neuroinflammation, and chronic pain are still not clear and the study of innovative approaches targeting neuroinflammatory pathways and their resolution is currently an emerging field of pain research. Dedicated to understanding this important and promising mechanism in chronic pain volume II of our Research Topic has built on the successes of volume I (Lione and Fisher, 2022) and includes four original research articles all focused on neuroinflammatory pathways in chronic constriction injury (CCI) rodent models of neuropathic pain. Here, we briefly introduce these publications in this editorial.

As described by Li et al., neuroinflammation is considered one of the prominent pathological mechanisms contributing to neuropathic pain. Neuroinflammation can arise from a variety of sources most notably via transcription/translation of inflammatory mediators from the nucleus of the cell which ultimately lead to activated microgliosis. Microgliosis is a central hallmark of neuropathic pain where the amoeboid activated proinflammatory M1 phenotypic state has been implicated in the development and progression of neuropathic pain (Karavis et al., 2023). Peroxisome proliferator-activated receptor gamma (PPAR γ), a member of a large group of nuclear receptors controlling the proliferation of peroxisomes has been shown to have anti-inflammatory functions in several macrophagous cell types (references in Li et al. via inhibition of microglia activation and ultimately reduction in neuroinflammation). The authors propose a novel mechanism of action by which signalling through PPAR γ may produce both an anti-inflammatory and analgesic action via inhibition of the M1 phenotype (the major inflammation promoting microglia) and the CX3CR1 signalling pathway in spinal microglia in rats with a sciatic nerve CCI thereby offering a novel therapeutic treatment approach for the management of neuropathic pain.

Huang et al. have demonstrated that whereas the pro-inflammatory signal transducer and activator of transcription 3 (STAT3) is increased following nerve injury by contrast STAT2 is reduced in rats with a sciatic nerve CCI neuropathic pain. Huang et al. demonstrate that reduction of the nuclear distribution of STAT2 induced microglial activation leading to an increase in major histocompatibility complex class II (MHC II) protein in the spinal dorsal horn and production of pro-inflammatory factors. Increases in MHC II consequently drive immune responses to extracellular and intracellular stimuli and ultimately the transcription of pro-inflammatory genes. Transcriptional regulators within the cell provide another potential site for controlling the production of both pro- and anti-inflammatory genes and thereby a potential target for the treatment of chronic pain and the Huang et al., study supports that restoration of nuclear expression of STAT2 could be a potential pathway for the treatment of neuropathic pain.

Choi et al. also examined the modulation of neuropathic pain in a mouse model of sciatic nerve CCI by exploring the neuroprotective and putative analgesic effect that the neurosteroid progesterone and the metabolic enzyme of progesterone (P450c17) may possess on neuroinflammatory glia cells such as astrocytes. They demonstrated that the temporal profile of progesterone is crucial to the presentation of analgesic activity. For example, administration of progesterone during the early phase of chronic pain acts to facilitate mechanical allodynia as well as the pathological activation of astrocytes via activation of P450c17. Whereas progesterone administration during the maintenance phase of chronic pain has an analgesic effect of the development of allodynia and an inhibitory effect on astrocyte activation. The authors conclude that the important role of P450c17 in mediating the effects of progesterone should be considered in order to develop a more efficacious progesterone therapy for neuropathic pain maintenance control. This supports previous reports that astrocytes participate in the maintenance rather than initiation of neuropathic pain (Gosselin et al., 2010).

Although numerous molecular targets in the trigeminal ganglion (TG) and trigeminal subnucleus caudalis (Sp5C) have been identified currently, there are no consistently effective treatment options available for the rare chronic neuropathic pain condition trigeminal neuropathic pain (TNP) (Nagakura et al., 2022). Whole transcriptomic sequence analysis of the TG and Sp5C in a mouse model of neuropathic pain, the infraorbital nerve chronic constriction injury model (IoN-CCI), was coupled with the sophisticated use of bioinformatics to determine the biological pathway in the elegant study described by Cui et al.Examination of non-coding RNAs (ncRNAs) which have been previously linked to controlling mRNA expression during NP progression (Song et al., 2020) and TNP (Dong et al., 2022) revealed a high correlation with pain-related differentially expressed genes in the TG and Sp5C to anxiety, depression, apoptosis but, most importantly within the context of this editorial, inflammation and neuroinflammation. These findings indicate the involvement of ncRNAs in TNP development and open new avenues for research and treatment.

Together, the articles included in volume II of this Research Topic describes recent findings targeting microglia, astrocytes, and ncRNAs in modulating neuroinflammation pathways in rodent CCI models of neuropathic pain, providing valuable insights into potential therapeutic strategies for chronic pain.
